# Applied Bioinformatics and Public Health Microbiology: challenges, discoveries and innovations during a pandemic

**DOI:** 10.1099/mgen.0.000757

**Published:** 2022-01-28

**Authors:** Edward Cunningham-Oakes, Hannah Trivett

**Affiliations:** ^1^​ Health Protection Research Unit in Gastrointestinal Infections, HPRU Project Team, University of Liverpool, Ronald Ross Building, 8 West Derby Street, Liverpool L69 7BE, UK; ^2^​ Infection Biology and Microbiomes, Institute for Infection, Veterinary and Ecological Sciences, University of Liverpool, Leahurst Campus, Neston, Wirral, CH64 7TE, UK

**Keywords:** bioinformatics, pandemic, antimicrobial, resistance, COVID-19, SARS-CoV-2

## Abstract

The eighth Applied Bioinformatics and Public Health Microbiology (ABPHM) conference showcased the recent acceleration of bioinformatic approaches used in public health settings. This included approaches for the surveillance of infectious diseases, understanding microbial evolution and diversity and pathogen interactions. Overall, the meeting highlighted the importance of data-driven approaches used by scientists during the COVID-19 pandemic.

**Impact Statement**

This manuscript provides a detailed overview of the 2021 Applied Bioinformatics and Public Health Microbiology (ABPHM) conference, which showcased the recent acceleration of bioinformatic approaches used in public health settings. This included approaches for the surveillance of infectious diseases, understanding microbial evolution, diversity and pathogen interactions. The manuscript also contextualizes talks by providing insights and opinions as to the future of bioinformatics in public health.

## Introduction

The eighth Applied Bioinformatics and Public Health Microbiology (ABPHM) conference showcased the recent acceleration of bioinformatic approaches used in public health settings. This included approaches for the surveillance of infectious diseases, understanding microbial evolution and diversity and pathogen interactions. The conference was well balanced, with all novel bioinformatic tools having clear application to public health. The virtual conference format excelled in making the talks accessible to all delegates, with the ability to catch up on demand. This should be considered for future events to increase the number and broaden the diversity of attendees. Overall, the meeting highlighted the importance of data-driven approaches for outbreak management and surveillance used by scientists during the COVID-19 pandemic. Approaches described encompassed SARS-CoV-2, antimicrobial-resistant pathogens, and the use of parallel, non-genomic data streams to provide robust epidemiological surveillance.

### Keynote

The conference began with **Dr Maria Van Kerkhove** from the World Health Organisation (WHO) noting that researchers have achieved rapid catalysis of sequencing capacity that the WHO have strived for, thanks to their ongoing efforts during the pandemic. Catalysis has been made possible by utilizing pre-existing pathogen genomics resources such as the Global Influenza Surveillance and Response System (GISRS). There is however a need to develop surveillance systems for a variety of pathogens to avoid continued, unsustainable leverage of the GISRS. To be fit for purpose, these systems need to be able to respond to future variants of concern and pandemics by sequencing intelligently, and subsequently achieving better geographic representation with comprehensive metadata. The public health research community should be aiming for a sustainable, global network of infectious disease surveillance, with coordinated reporting. The foundations for other key themes that would be echoed by speakers throughout the conference were provided by **Dr Ahmed Ouma**, the Deputy Director of the Africa CDC. This keynote detailed the African pathogen genomics initiative and emphasized the need for genomic sequencing infrastructure to be more accessible in low-middle income countries (LMICs) for viable pathogen surveillance and outbreak management. It was also noted that partnership between public and private sectors globally could help to surmount these challenges by improving logistics of transport between borders, accessibility to the market and providing time-relevant training for individuals within the laboratories [1].

### COVID: lessons learned and pandemic preparedness

The talks to follow served to accentuate the message of the keynote speakers, by providing robust evidence for the indispensability of genomics during the pandemic.

**Professor Edward Holmes (University of Sydney, Australia**) showed that metagenomics has been crucial for the detection of new viruses and to identify co-infections within patients with pneumonia, including influenza, parainfluenza, SARS-CoV-2 and diverse bacteria in different abundances [2]. This talk highlighted the importance of fast metagenomic sequencing for diagnostics to inform patient treatments and the utility of metagenomics for the surveillance of infectious diseases. Surveying people living and working at the human–animal interface was highlighted as an effective method to prevent future pandemics from occurring. The value of this type of integrated surveillance that accounts for human–animal interactions is already widely acknowledged under One Health [3]. Integrated surveillance would also inform public health agencies about circulating pathogens and the emergence of new variants [4].

In the next session, **Professor Benjamin Howden (Doherty Institute, Australia**) spoke about the use of genomic data to inform hotel quarantine procedures as a method of controlling viral transmission. The efficacy of these procedures was demonstrated via the eradication of genomic clusters. The recognizable value in this data has highlighted the need to facilitate improved public health genomics data sharing and analysis. This has led to the development of tools such as AusTrakka [5] by Torsten Seemann and Anders Goncalves da Silva, enabling real-time analysis of SARS-CoV-2.

**Dr James Shepherd (MRC – University of Glasgow Centre for Virus Research, UK**) provided an insight into how specific mutations may result in SARS-CoV-2 variants with greater potential for pathogenesis and transmission. The N439K variant was described [6], with enhanced binding affinity to the hACE2 receptor and resistance to monoclonal antibodies. This work linked closely to the final talk of the session by **Dr Tanya Golubchik (University of Oxford, UK**) who discussed how genome sequencing facilitated a greater understanding of SARS-CoV-2 transmission, including the diversity of variants within hosts and transmission routes. The research presented provided a wider understanding of the diversity of SARS-CoV-2 with regard to transmission and viral evolution [7].

The talks drew on the knowledge gained from the mobilization of genome-sequencing taskforces to understand the pandemic and supplied scope for future applications of genomic surveillance. Each talk offered a different approach utilizing genome sequencing as an aid to disease surveillance, which could have the potential to benefit public health agencies by adding tools to the current armoury for tracking disease transmission. Overall, speakers highlighted that the use of targeted and non-targeted metagenomic sequencing is invaluable to identify pathogens of interest for surveillance. Both approaches will be crucial for the management of future outbreaks.

### Environmental genomics for public health

The second session provided further applications of SARS-CoV-2 genomics, alongside several studies demonstrating important advances in bacterial genomics, both for profiling antimicrobial resistance (AMR) and exploiting metabolic potential.

The first talk presented by **Dr Ameet Pinto (North-eastern University, USA**) noted current drinking water surveillance data has limited public availability. A recent study comparing AMR gene profiles (or resistomes) in disinfected and non-disinfected drinking water systems was then described. The study showed that water resistomes were distinct to each system with lower taxonomic diversity in disinfected systems. Within the disinfected system, the microbiome was found to have a higher proportion of glyoxylate shunt pathways, and pathways for reuse of fatty acids, suggesting that microbiomes in these systems may be better adapted to use the available biomass [8]. These findings will help to inform water agencies as to how they may minimize entry of undesirable microbes into water systems.

**Dr Matt Olm (Stanford University, USA)** then presented inStrain, a tool which uses the novel metric, population Average Nucleotide Identity (popANI). By leveraging nucleotide diversity, linkage disequilibrium, identification of single-nucleotide variants and coverage breadth and depth calculations, the user can characterize the population-level diversity [9] of each detected micro-organism within a metagenomic population. inStrain has far-reaching applications, as demonstrated by its application to >1000 infant faecal metagenomes for microbiome analysis [9]. This use case identified the diversity and origins of *Klebsiella *, depending on whether babies were delivered vaginally or by caesarean section [9].

**Catherine Pratt (University of Nebraska, USA**) evaluated the risks involved of returning to in-person learning during the pandemic using case studies from Nebraska Schools. Individual student PCR tests and wastewater sample data were combined to show the prevalence of SARS-CoV-2 across the region. As such, it was possible to identify high-risk activities such as choir practice and minimize the person-to-person transmission of the virus [10].

The final talk of the session by **Jenna Swarthout (Tufts University**, **USA**) demonstrated the use of short- and long-read sequencing data to uncover reservoirs of AMR genes within urban informal settings. It was highlighted that hotspots of said genes in soil and water provide a new hypothesis for the disproportionate growth of AMR in LMICs where these informal settlements are present [11].

Overall, the session illustrated that environmental surveillance is a powerful tool that can be used to track and understand pathogen transmission. Surveillance can also provide evidence for public health agencies to pragmatically instate policies and conduct public health enquiries. One of the highlights of the session was the discussion around a need to standardize prediction of AMR from genomes in public health. Certain projects are currently aiming to address this via a harmonized approach between multiple databases (see https://github.com/pha4ge/hAMRonization).

### Global public health genomics

Session three showcased the advances in genomics applied to problems faced in LMICs. Two separate investigations into the AMR of *Salmonella typhi* by **Dr Senjuti Saha (Child Health Research Foundation, Bangladesh**) and **Dr Philip Ashton (Malawi-Liverpool-Wellcome Unit, Malawi**) showed that (i) azithromycin resistance can arise spontaneously in extensively drug-resistant *S. typhi* (which is only sensitive to this oral antibiotic) [12], and (ii) the emergence of fluoroquinolone resistance in *S. typhi* was attributed to multiple QRDR mutations, with increased prescription of fluoroquinolones providing selective pressure [13]. Both talks highlighted the need for antimicrobials to be developed in the face of the current AMR crisis sweeping across the globe.

**Dr Sonia Sia (Research Institute for Tropical Medicine – Department of Health, Philippines**) emphasised the need to tackle the global AMR crisis by presenting recent studies on exploiting genomics to profile the AMR in *Shigella flexneri* serotypes. The aim of the study was to understand the AMR and clonality of *S. flexneri *, the second most frequently identified diarrheal pathogen in the Philippines. In a panel of 55 isolates, 40 % were phenotypically MDR to ampicillin, cotrimoxazole and chloramphenicol. Additionally, ten isolates were then fully sequenced, and were determined to comprise two sequence types, with one variant at the *fumC* locus constituting the difference between the sequence types. For all fully sequenced isolates, the predicted *in silico* AMR profile reflected their phenotypic MDR.

The final speaker, **Ifeoluwa Akintayo (University of Ibadan, Nigeria**), reinforced the importance of having a solid foundation of microbiology capability in rural areas, such as laboratories capable of genome sequencing to deliver faster diagnostics. The ability to provide a more comprehensive treatment plan, specific to the predicted AMR profile of the isolated pathogen would allow for better treatment of patients in rural areas, where pathogen-specific treatment is currently unavailable.

The session concluded with a question-and-answer session where all individuals echoed the looming threat of AMR in LMICs, and the need to improve capabilities of international laboratories in LMICs to combat this. These improvements will only be achieved by investing in infrastructure, training and resources. Once this baseline is achieved, fully resourced microbiology laboratories can be developed and integrated to suit the local public health needs.

### Bioinformatic showcase

Novel bioinformatic approaches are commonly developed as a basic science interest, to improve upon earlier approaches, or because no previous approach exists to solve a biological problem [14]. One common issue in the modern era is the management and analysis of the vast quantities of data available. This issue is driven by the generation of new data, and augmented by the ability to more readily share data that is already available [15].

As a testament to this, the numerous public health challenges posed over the past year have resulted in a data explosion, all of which has been effectively used to help pandemic response.

[16]. The bioinformatics showcase provided insights into how the current public health arsenal is being bolstered with novel bioinformatic approaches. The session provided new algorithms and bioinformatic workflows, which are easy to use, accessible, cost effective and have the potential to provide data which is time relevant, and patient focused.

The talks began with **Peter van Heusden (South African National Bioinformatics Institute, South Africa**) who presented his work on the COMBAT-TB workbench [17], a high-throughput web-based tool for routine *Mycobacterium tuberculosis* analysis, accessible to non-specialist users. This tool improves the disjointed nature of using individual programmes for genomic analysis, and provides a comprehensive breakdown of data, using visual aids such as graphs. COMBAT-TB also provides proof of principle for accessible web-based analysis, with easy-to-understand graphical interfaces. This same principle could be applied to other pathogens and would enable analyses to be performed by public health workers with varying informatic ability.

**Verity Hill (University of Edinburgh, UK**) highlighted CIVET [18], a recent bioinformatic innovation in SARS-CoV-2 research. This real-time genomics tool enables sample comparison to reference phylogenies and merging of samples into the same phylogeny, without the need to recreate a new phylogeny each time a new sample is added. CIVET uses metadata to create a comprehensive report for outbreak investigations and summarizes SARS-CoV-2 strain diversity in the context of strains from the selected geographical range (global or UK-wide analysis). The ability to subset by geographical range enables data to be provided in a time-relevant manner for the increased volume of SARS-CoV-2 genomes being submitted into CIVET.

**Dr Bede Constantinides (Nuffield Department of Medicine, UK**) presented Konstel [19], which addressed biological namespace saturation by generating hash-based, unique, pronounceable and memorable names. This command-line tool minimizes difficulty in sharing restricted data during the emergence of a novel outbreak, but still allows for researchers to compare similarity using phenomic identifiers. Notably, since the conference took place, the WHO has adopted a similar approach, using Greek lettering for variant nomenclature.

The final talk of this session was presented by **Samuel Horsfield (Imperial College London, UK**) who provided an in-depth explanation of a gene caller for bacterial pangenome graphs. Graph Gene Caller (ggCaller) [20], exploits the representation of population genetic variance within pangenome graphs and enables accurate gene calling and reference-free variant calling directly from WGS reads. Future developments for this tool look to improve scalability.

The tools presented in this session provided many welcome additions to our armamentarium against microbial pathogens. It was recognized however that there is a current gap in bioinformatics training, which can make obtaining and installing tools from package managers difficult for a non-specialist user. Whilst the development of graphical user interfaces is aiming to make bioinformatics more accessible [21], the value of cross-discipline training can be overlooked (but has been commented on elsewhere [22]). The presence of a specialist can circumvent any gaps in knowledge that may hinder individuals within a team of collaborators. However, as a community, we should work towards developing communication and integration of disciplines, and indeed training, as improved understanding of expertise would significantly narrow these gaps across all avenues of scientific research.

### Digital epidemiology

To keep innovating within public health, it is essential to decompartmentalize infectious disease management, which is traditionally divided into (i) individual testing and clinical management, and (ii) epidemiology and public health microbiology. One historical reason for compartmentalization has been the lack of digitization of epidemiological data. Digital epidemiology is broadly defined as epidemiology data that has been digitized [23]. In the context of bioinformatics and public health, this presents a unique opportunity to integrate genomic and epidemiological data streams. The digital epidemiology session provided important insights on how to best make use of non-genomic data streams to aid public health.

**Dr Emily Turner (Gates Foundation, USA**) described the use of digital tools to avoid errors in administration of testing and interpretation of test results, as well as to reduce omissions in reporting. The COVID-19 pandemic has made it essential to develop ‘closed-loop systems’ linking epidemiological data with community-based testing, though the value of this is not limited to COVID-19 alone. Digital health interventions identified in this process included timers, digital guidance notes and videos for image capture and interpretation. This highlights that digital services and data pipelines are key enablers to connect diagnostic testing with public health. The Seattle Flu Study was also highlighted as an example of best practices when implementing this type of approach in infectious disease management [24].

**Dr Maimuna Majumder (Harvard University, USA**) described advanced computational epidemiology involved in the manipulation of large, diverse datasets and how diseases function within human populations. Data was then discussed from three papers, which (i) showed that salutary sheltering and social distancing reduces infections and deaths due to COVID-19 [25]; (ii) showed that, in contrast to other coronavirus diseases resulting from MERS-CoV and SARS-CoV-1, laboratory research of COVID-19 was underrepresented compared with clinical, field-based and modelling data. This was principally due to insufficient funding for basic research [26]; (iii) investigated the effects of former (U.S.) President Trump’s public suggestion that injection of disinfectant should be investigated by the White House Coronavirus Task Force, despite regulatory and public health organizations advising against the practice [27].

**Dr John Lees (Imperial College London, UK**) presented an array of bioinformatics tools that provide routes for public health data generators to use and analyse sequences in real-time (<24 h): (i) Pp-sketchlib a replacement for Mash (2000× to 3000× faster), (ii) PopPUNK, an MLST replacement with improved clustering, which visualizes distances for phylogenetic analyses, (iii) PopPUNK-web, a web-based version of PopPUNK, which eliminates the need to download or upload data, enhancing usability and data security (currently only *Streptococcus pneumoniae* sequences are supported) and (iv) PopPIPE, a scalable, reproducible, automated pipeline, that subclusters each PopPUNK cluster and creates maximum-likelihood trees to provide greater resolution [28].

A final talk by **Dr Sion Baylis (University of Bath, UK**) described how machine learning can be used for the epidemiological tracking of (10 000) *Salmonella enterica* subspecies Enteritidis, which undergoes routine surveillance by the UK Health Security Agency. A proxy, known as the SNP address [29] was found to be effective for epidemiological tracking. SNP address enables real-time cluster detection by providing a discrete seven-digit code. Each digit represents an SNP distance in comparison to a reference genome at a different SNP distance threshold. However, this means that any additional genomic characterization, such as an accessory genome analysis [30] is not provided. Precision was improved in sub-continental regions by capturing larger amounts of genomic variation using unitigs, contigs that are fully consistent with all data, including mate constraints, and reads. It was noted that this method could be used for other pathogens, and smaller-scale data sets in local regions, so long as representative, good-quality samples with sufficient metadata are available.

### Late-breaking science

In the final session of the conference, speakers introduced the latest applications of genome sequencing and bioinformatics that have the potential to be embedded into public health policy informing and disease management infrastructure.

**Professor Stephen Bentley (Wellcome Sanger Institute, UK**) described the Global Pneumococcal Sequencing Project (GPS) [31], one of the largest pathogen genomics projects undertaken to inform vaccine development against *Streptococcus pneumoniae *. The aim of the project was to sequence over 20 000 *S *. *pneumoniae* strains using WGS by 2020 to provide information on AMR profiles, strain type and capsule type, whilst also documenting relevant information from the metadata such as the geographical origin. This information is key to inform vaccine design to safeguard against Pneumococcal diseases in the future. The talk highlighted the rise of serotypes not targeted by the vaccine, such as the 24F serotype. Notably, this talk also provided a unique case study for providing bespoke support for collaborators in LMICs, thus synergizing with the messages of both the keynote, Dr Ahmed Ouma, and Ifeoluwa Akintayo in the late-breaking science section. As described in the associated publication, one of the fundamental challenges in developing a surveillance system that can be applied to LMICs, is variation in local infrastructure and resources. Recognizing this, alongside individual constraints and motivations will enable the public health research community to collaborate more effectively to implement accessible surveillance [32].

**Dr Koji Yahara (National Institute of Infectious Diseases, Japan**) described the utility of long-read metagenomics to provide much-needed characterization of the human saliva microbiome, which identified phages and jumbo phages with homologues of AMR genes using PromethION sequencing technology. This study was also able to identify novel phages, and clearly place them in host genomic context. This study provided a clear example of the utility of long-read sequencing to improve viral genome recovery from the oral microbiome, which can suffer in the absence of longer reads [33]

SARS-CoV-2 was revisited by **Darlan Da Silva Candido (University of Oxford, UK**) who provided an overview of the emergence of SARS-CoV-2 lineage P.1 in Brazil [34]. A resurgence in SARS-CoV-2 infections occurred in late 2020, owing to the acquisition of 17 mutations including a trio in the spike protein (K417T, E484K and N501Y). The exhibited mutations carry the potential for increased transmissibility and immune evasion leading to increased mortality where these variants are present. Recently, lineage P.1 has been renamed as the gamma variant by WHO, again demonstrating a need for systematic nomenclature, first highlighted by Dr Bede Constantinides during the Bioinformatic Showcase. This talk also suggested that providing non-pharmaceutical intervention whilst vaccine programmes were established, coupled with real-time surveillance to track variant frequency had the potential to reduce the circulation of new variants, which is being studied further.

**Dr Nicole Wheeler (University of Birmingham, UK**) introduced the development of novel computational methods using genome sequencing and machine learning for the Nuclear Threat Initiative (NTI) based at the University of Birmingham. The project aimed to prevent the procurement of DNA sequences of potential public health risk without adequate clearance. Moreover, the tool offered scope to understand the genetic changes of pathogenic microbes by building a bio-risk database that can be used to screen DNA against known lists of microbial genomes and virulence genes to identify emerging biological threats. Whilst the development of such screening tools provides utility for the NTI, it also offers potential applications for use with circulating infectious agents, which may have variants that pose a threat to public health, providing a concept for pathogen surveillance that could improve public health disease management in the future.

**Carla Mariner-Llicer (Instituto de Biomedicina de València-CSIC, Spain**) presented a talk investigating the feasibility of direct WGS of *Mycobacterium tuberculosis* [35], compared with conventional culturing methodologies, which are time consuming and labour intensive. The study found that all clinical specimens selected for downstream analysis (28/37) clustered with their matching culture isolates. Direct WGS offers the possibility to provide culture-independent surveillance of *Mycobacterium tuberculosis *, identifying drug susceptibility and transmission inferences promptly, providing information for treatment in a relevant timeframe. This study offered scope for real-time epidemiology surveillance and to identify drug resistance in locations where it is not feasible to set up a clinical microbiology laboratory.

### Concluding remarks

ABPHM brought together an international community of scientists specializing in sequencing and epidemiology and highlighted the recent successes of integrating bioinformatic approaches into public health microbiology. Delegates and speakers demonstrated the potential of novel data-driven genomics in meeting the needs of public health agencies. These needs are principally; (i) understanding the spread and evolution of pathogens, including any underlying causes [36]; (ii) monitoring the development of AMR in pathogens of interest [37]; (iii) the continuous development of readily accessible tools and workflows for both obtaining and manipulating data from both genomic, and non-genomic sources [3, 23].

One of the major overarching themes of the conference was the need for integrated surveillance and early detection. The talks presented at ABPHM contribute significantly to this need, where an ideal integrated surveillance system has been highlighted as one that promotes active surveillance of diseases in such a way that enables rapid response to an outbreak. This approach should be underpinned by portable genome sequencing and digital epidemiology, using a ‘One Health’ approach to account for pathogen reservoirs from human, environmental and animal niches [3]. As demonstrated by our response to the pandemic, genome sequencing has provided evidence of capability to do this, but is impacted by factors such as cost, technology development and being received in scientific community [3, 38]. However, SARS-CoV-2 sequencing infrastructure was developed in response to the pandemic, rather than in anticipation. Moreover, this infrastructure leverages our existing resources to the extent that routine surveillance of other diseases has suffered [39]. As such, future work should be geared towards more agnostic genomic diagnostics for infectious disease, that are primed to respond to unusual disease outbreaks, as well as well-characterized. This would serve to both improve our ability to detect novel outbreaks and lessen compromisation of ongoing diagnostics [40]. Challenges exist in this approach, due to the inherent lack of a known universal target using non-specific approaches and determining correct interpretation and actionability of results [41]. If these technical challenges can be overcome, this approach could be promising for the future of genomic surveillance.

To ensure the gap between LMICs and others within the research community is not further increased, there needs to be an improved capacity for data sharing, which overcomes accessibility barriers experienced by LMICs [37], whilst also accounting for concerns about exploitation and data-sharing raised by LMICs. Such concerns include data inequality stemming from contribution to large data repositories, whilst being unable to perform big-data analyses owing to a lack of resources [42]. This data inequality has the potential to prevent LMIC researchers from seeing the benefit of large grants and top publications [42]. By improving accessibility, health organizations and clinical researchers would stand to benefit as the work would not be isolated in areas that are resource rich. Distributing resources and funding would improve accessibility, equitably improving public health research and implementation [43]. This will also contribute significantly to the overarching need for a sustainable genomic framework [36], emphasized by both keynote speakers. With the next conference scheduled in 2023, it will be exciting to see how the COVID-19 pandemic has paved the way for further developments and the successful implementation of bioinformatics in public health settings.
